# Why forests can mitigate floods of all sizes: Evaluating the scientific basis for forest-based flood mitigation

**DOI:** 10.1007/s13280-026-02346-6

**Published:** 2026-02-01

**Authors:** Samadhee Kaluarachchi, Younes Alila

**Affiliations:** Forest Resources Management, Faculty of Forestry and Environmental Stewardship, The University of British Columbia, 2424 Main Mall, Vancouver, BC V6T 1Z4 Canada

**Keywords:** Causal inference, Flood management, Forests, Nature-based solutions, Stochastic methods

## Abstract

Rising flood frequencies and magnitudes are causing the loss of lives, infrastructure damage, and economic disruption, making effective flood management imperative. Nature-based solutions are increasingly promoted, but whether forests mitigate large floods remains a decades-long scientific controversy. Resolving this issue is crucial, as flood management necessitates reliable projections and a sound, scientifically defensible understanding. We show that the conventional deterministic approach uses a non-relevant research question, improper hypothesis, and non-causal experiment, rendering its conclusion that forests have little impact on large floods scientifically indefensible. In contrast, a causal, stochastic approach captures the probabilistic nature of floods and allows for studying anthropogenic effects to hydroclimatic variables through its relevant research questions, falsifiable hypotheses, controlled experiment, and sound physical reasoning. The defensible stochastic approach must replace flawed alternatives, suggesting that forests *can* mitigate large floods. With rigorous methods, hydrology can support strategies which meaningfully reduce flood risk in an increasingly uncertain future.

**Supplementary Information:**

The online version contains supplementary material available at 10.1007/s13280-026-02346-6.

## Introduction

### The rise of nature-based flood mitigation

Increasing flood frequencies and magnitudes worldwide (e.g., Hirabayashi et al. 2021; Gillett et al. 2022) are causing the loss of human and non-human lives, damaging infrastructure, provoking lawsuits, and disrupting key economic sectors (Newman and Noy 2023). These impacts have intensified debate over underlying drivers of these extremes and who should be held accountable. In both scientific (e.g., Hirabayashi et al. 2021; Gillett et al. 2022) and policy-making arenas (Lahsen and Ribot 2022), anthropogenic climate change is often considered the main driver of large flood events. At the same time, failing to acknowledge the role of land use in amplifying risk can divert attention from feasible land use and land cover (LULC) management by local and national governments (Lahsen and Ribot 2022). Overly emphasizing climate as the sole cause can shift the focus away from policy initiatives that could have delivered local remedial action (Lahsen and Ribot 2022) and overlook the potential of land use management to mitigate flood vulnerabilities, especially under increasing climate-related risks. Thus, the roles of both climate and LULC changes must be examined through scientifically robust methods to understand changing risks and implement cost-effective solutions.

Solutions for flood mitigation are increasingly drawing from nature. Terms such as nature-based solutions (NBS) advocate for holistic flood mitigation while providing multiple socioeconomic benefits and ecosystem services (Dadson et al. 2017; Lallemant et al. 2021; Yu et al. 2025). They generally aim to increase catchment storage capacity and temporarily store precipitation, reducing peak discharge (Penning et al. 2023). Many initiatives include maintaining or increasing forest cover, based on the understanding that forests evaporate and transpire precipitation back into the atmosphere, enhance soil infiltration, and promote groundwater recharge that sustains dry season baseflow (Hurkmans et al. 2009; Penning et al. 2023). Consequently, NBS have been, or are being, implemented in many countries worldwide (e.g., Faivre et al. 2018; Jongman 2018).

### Scientific perspectives on nature-based flood mitigation

Despite increasing public support for NBS, forest hydrology remains heavily opposed, or at least divided, on the effectiveness of forest cover on flood mitigation, especially for large (i.e., > 10-year) events (Stratford et al. 2017; Environment Agency 2018). This divide originates from decades of studies investigating how a change in forest cover alters downstream floods (e.g., Bathurst et al. 2011; McEachran et al. 2021). Many studies adopt a Before-After, Control-Impact (BACI) approach, where two neighboring watersheds of similar physical and climatic characteristics are monitored prior to disturbance and their streamflow is calibrated (e.g., Bathurst et al. 2020). The treatment watershed is then disturbed (e.g., harvested), while the control remains undisturbed. In most analyses, observed streamflow peaks in the control watershed are used to estimate peaks had the treatment not occurred. Estimated peaks are compared with harvested watershed peaks using one of two primary analysis methods for understanding and predicting floods (described in the following paragraphs). Conclusions regarding the effects of forest cover on floods, especially for large and extreme events, inform scientific perspectives on the use of forests for flood mitigation.

Forest hydrology is mostly skeptic of the mitigative effects of forest cover on large floods (e.g., Dadson et al. 2017; Xiao et al. 2022), shaped largely by conclusions from the “deterministic” approach dominating forest hydrology. When analyzing BACI experimental results (or results from a modeled catchment with multiple scenarios of varying forest cover), deterministic studies define the effects of changing forest cover on flood magnitude as the difference in event magnitude between control and treatment watersheds subject to the *same storm or snowmelt event* (hereafter referred to as the “deterministic” approach in forest hydrology). Although these studies rarely directly address extreme events (DeWalle 2003), they conclude that forest cover mitigates small and medium floods but cannot mitigate large events (e.g., Xiao et al. 2022; Barnes et al. 2023). Many even suggest an event size “threshold” beyond which forest removal has little or no effect on floods, which ranges from as low as the 2-year flood event (Thomas and Megahan 1998; MacDonald and Stednick 2003) to more commonly the 5- to 10-year event (Bathurst et al. 2011, 2020; Birkinshaw et al. 2011). Deterministic studies have been conducted in small to large catchments over many hydroclimatic regimes including rain, rain-on-snow, and snow environments around the world (e.g., Guillemette et al. 2005; Bathurst et al. 2020 and citations therein) (see Supplementary Table S1), fueling skepticism toward forest-based solutions for mitigating large floods (DeWalle 2003; Dadson et al. 2017; Xiao et al. 2022).

This scientific skepticism is echoed in several influential policy papers (e.g., Stratford et al. 2017; Environment Agency 2018), with some suggesting that the idea of forests mitigating (large) floods is manufactured by the media and conservationists with little scientific backing (CIFOR and FAO 2005; Calder and Aylward 2006), possibly as a “red herring” to oppose development (DeWalle 2003, p. 1256). With this idea “far more widespread in *public* opinion than in *scientific* circles” (Xiao et al. 2022, p. 7), there seems to be a disconnect between scientific and public perceptions and between scientific perception and policy (DeWalle 2003; CIFOR and FAO, 2005; Calder and Aylward 2006; Xiao et al. 2022).

Results from a less dominant approach within and outside of forest hydrology challenge deterministic conclusions. “Stochastic” studies draw on probability concepts which capture the stochastic nature of floods (Klemeš, 1978). When analyzing BACI experimental (or modeled) results, they instead define effects of changing forest cover as the difference in peak flow magnitudes which are *of the same frequency* between the control and treatment watersheds (hereafter referred to as the “stochastic” approach). Alternatively, they investigate how a change in forest cover changes flood frequencies for flood extremes *of the same magnitude*. Stochastic studies have been conducted across small to very large catchments in varied hydroclimatic regimes (see Table S1) (e.g., Hurkmans et al. 2009; Birkinshaw et al. 2011; McEachran et al. 2021), collectively suggesting that forests have the potential to mitigate small, medium, *and* large floods.

### A scientifically defensible approach

Persisting scientific controversy and skepticism has not prevented the promotion or implementation of NBS including forests, either acknowledging this skepticism (e.g., Penning et al. 2023) or not (e.g., Yu et al. 2025). In fact, the forests and floods relationship remained a point of contention among foresters, engineers, conservationists, and the public for centuries, centering on the extent to which deforestation increased flood risk and the role of forests in flood management (Saberwal 1998). Today, proponents of NBS continue to maintain the intuitive claim that forests mitigate floods. Could they deserve more credit than given by the scientific community (Bruijnzeel 2005)?

While some argue that both approaches can co-exist (Bathurst 2014; Birkinshaw 2014), policymakers cannot enact effective solutions with contradictory scientific advice. Science must present clear guidance as to whether forests play a role in flood mitigation. This requires examining which conclusion(s) can be supported based on the suitability of each framework for understanding and predicting flood extremes. Are both approaches equally rigorous, or must one be abandoned?

Ultimately, effective flood management hinges on accurate flood prediction, as human activities varying over space and time modify the climate and LULC. Reliable prediction requires a sound understanding of the relationships between anthropogenic drivers and floods, which is also key for informing flood management. A causal, *scientifically defensible* understanding of the forests and floods relationship in a changing climate is crucial.

To ensure that reliable science guides policy and management, this paper assesses deterministic and stochastic literature examining the effects of changing forest cover on flood peaks, advocating for a scientifically rigorous approach. The Scopus database was used to search for appropriate literature using keywords such as “forest,” “harvest,” “flood,” “peak,” and “hydrology” with “*” and the Boolean operators “AND” or “OR” as necessary. Titles and abstracts were examined for whether they quantitatively investigate the effects of changing forest cover on floods using a deterministic or stochastic approach, then full texts of relevant studies were examined. Studies which investigated other hydrological variables (e.g., water yield) without any connection to peak flows were discarded such that only those linking forest cover changes to flood peaks remained. Studies were examined for their typical research questions, hypotheses, experimental designs, and results. The strengths and limitations of each approach are discussed by incorporating key insights from wider hydrology and climate science.

Section "Causally investigating effects of forest cover changes on floods" lays out the causal framework needed to disentangle how different anthropogenic activities are changing floods. Section "Evaluating two scientific approaches to the forest and floods disagreement" examines the suitability of the underlying research question, hypothesis, and experimental design of both frameworks to answer whether forests can mitigate floods. Finally, Section "Revisiting the role of forests in flood management" reframes the dispute, highlighting which conclusion(s) are scientifically rigorous and should inform flood policy and management.

## Causally investigating the effects of forest cover changes on floods

### Causality is central to the science of extremes

Whether forest cover—rather than climate or other LULC changes alone—is a main driver of downstream flooding has received much attention. Policy reports criticize the public and media for oversimplifying “overlapping influences” of anthropogenic activities (CIFOR and FAO, 2005, p. 3). These same, if not higher, standards must also apply to science: Has its mainstream understanding emerged from a framework which causally isolates the effects of forest cover changes on floods?

In Earth systems science, multiple superimposing drivers act on the variable or system’s response of interest, convoluting cause-and-effect relationships (Runge et al. 2019). This is particularly pronounced in observed time series (e.g., streamflow records), where only a causal inference approach distinguishes cause-and-effect relationships amidst other drivers influencing the time series (Runge et al. 2019). Combined with their profound societal impact, the rarity (and consequent sampling errors (e.g., Bernardara et al. 2008)) of flood extremes makes causal attribution necessary for reliable insight. Identifying changes in forest cover as a cause of downstream flooding thus requires causally isolating the effects of forest cover changes on floods.

Identifying the extent to which anthropogenic activities causally modify hydroclimatic events, especially extremes, must be a shared pursuit across hydrology and climate science. Similar to how increased flooding prompted forest hydrology to investigate their drivers, increasing frequencies and intensities of hydroclimatic extremes inspired climate science’s specialized branch of attribution science (e.g., Stott et al. 2004). Attribution science attempts to causally link (or *attribute*) anthropogenic climate change to shifts in hydroclimatic regimes (James et al. 2019). They investigate how human activity has increased likelihoods and magnitudes for a single type of event (e.g., Stott et al. 2004) or for the entire distribution from small to extreme events (i.e., general attribution) (e.g., Kirchmeier-Young et al. 2017).

The ability of attribution science to causally identify drivers of changing flood regimes broadened its application beyond climate change (e.g., Hirabayashi et al. 2021; Gillett et al. 2022) to LULC changes such as urbanization (e.g., Prosdocimi et al. 2015) and forest removal (e.g., Johnson and Alila 2023). In fact, attribution is at the core of forest hydrology’s current dilemma; whether anthropogenic disturbances to forest cover have altered downstream flood regimes, especially for extreme events, is a question of attribution. With many blaming logging operations for perceived increases in flooding (Bathurst 2014) and the need to implement effective NBS, the role of forest cover (or removal) on flooding must be causally understood.

This raises a critical question: What is the appropriate scientific framework in forest hydrology to causally understand (and predict) changing flood regimes?

### What is the appropriate causal framework?

A strong scientific framework must capture key characteristics of the system under study. For floods, this involves capturing its *multiple* flood generation factors, each of which are stochastic (i.e., *random*) and *dynamically interacting* with each other.

Floods are natural phenomena which occur with or without climatic and LULC changes and are thus governed naturally by *multiple* controls. Rainfall input, snowpack accumulation, antecedent catchment wetness, and energy available for snowmelt are all climatic controls driving floods (e.g., Gillett et al. 2022). Physiographical controls modulate climatic influences by modifying above-ground (e.g., snowpack) and below-ground (e.g., within soils) storage of water, synchronizing or desynchronizing snowmelt throughout the watershed, and influencing the efficiency with which runoff is delivered to the channel network (Tonina et al. 2008; Kaluarachchi and Alila 2025). For example, deeper and permeable soils, larger catchment area, and the presence of water-retaining features such as lakes, wetlands, and ponds increase available storage capacity, promote deeper flowpaths, and attenuate runoff and hence flood peaks (Klemeš, 1970; Blöschl and Sivapalan 1997; Kusumastuti et al. 2007; Chen et al. 2021). Homogeneity or heterogeneity in slope aspect, elevation, and other environmental characteristics helps synchronize or desynchronize (respectively) timings at which stored water is delivered to the channel network, influencing runoff and hence exacerbating or attenuating (respectively) flood peaks (Duncan 1995; Ellis et al. 2011).

These *multiple* natural controls vary *randomly (i.e., stochastically)* in space and time. As climatic controls vary stochastically (e.g., spatiotemporal variation in energy and precipitation), the role of physiography in influencing the hydrological response also becomes stochastic. For instance, catchment storage in features such as lakes, wetlands, floodplains, and soils coinciding with a precipitation or snowmelt event is the legacy of past stochastic weather conditions and wetness states, thereby making catchment state stochastic (Robinson and Sivapalan 1997). Similarly, source areas and stream networks expand and contract as a response to precipitation inputs (Rogger et al. 2012; Basso et al. 2023); thus, they also vary stochastically in space and time. In this way, flood generation is shaped by the probabilistic (i.e., stochastic) nature of floods. Many key flood generation processes such as hydrological connectivity are best encapsulated at the macroscale (McDonnell et al. 2021); thus, spatiotemporal variability must be incorporated through causal, stochastic methods at appropriate scales.

Furthermore, stochastic climatic and physiographic controls interact with each other *dynamically* through space and time. Controls cannot be hierarchically organized by importance because any combination may amplify or attenuate streamflow (Johnson and Alila 2023; Pham et al. 2025). For example, in snow environments, annual flood peaks arise from any combination of snow accumulation and melt rates (among other controls). If science is to understand and predict floods, it must capture the *multiple, stochastic,* and *dynamically interacting* nature of its drivers.

This understanding is not unique to hydrology. First originating from quantum mechanics, viewing stochasticity as an inherent system property rather than a mere consequence of incomplete knowledge is now widespread across the sciences (Klemeš 1978). In quantum mechanics, this recognition shifted the focus away from deterministically predicting single measurements to probabilistically predicting outcomes from the entire set of measurements (Ballentine 1970; Loewer 2020). This shift contributed to a broader rethinking of causality and an interest in stochastic approaches across scientific disciplines, including hydrology (“stochastic hydrology”) (Klemeš, 1978). Stochastic hydrology captures the stochastic nature of floods and centers physical understanding into experimental design and interpretation to support scientifically sound analysis (Eagleson 1972; Merz and Blöschl 2008), although to our knowledge its connection to causal inference has rarely been discussed (e.g., Klemeš (1978)). Stochastic approaches are central to attribution studies (Allen 2003; Otto 2017; Swain et al. 2020) and other sciences, where they account for the “multiple and chancy” nature of phenomena (Karhausen 2000, p. 59) and are considered highly suitable for causal studies (e.g., Parascandola and Weed 2001; Loewer 2020).

In hydrology, the *multiple, chancy*, and *dynamically interacting* nature of flood controls suggests that an event of a given magnitude can be stochastically generated by many combinations of variables. For all events of the same magnitude (e.g., when stored water nears watershed storage capacity), combinations of controls differ. This is long understood in attribution science; any one event never occurs the same way again with its unique combination of drivers, but events of the *same magnitude* may reoccur with different combinations (Otto 2017; Philip et al. 2020). Prediction thus requires shifting away from a deterministic focus on singular events to a stochastic focus on classes of events (i.e., quantiles) to assess whether event magnitudes are changing (Otto 2017). Furthermore, as magnitude and frequency are “two sides of the same analytical coin” (Swain et al. 2020, p. 525), any event of a given magnitude occurs with its corresponding frequency. Event classes thus represent the various stochastic combinations in which events of a given magnitude and frequency are generated.

Both magnitude *and* frequency must be evoked to understand how anthropogenic activity influences flood quantiles. High-magnitude events may still occur by chance without human-mediated changes, but human activities change their likelihoods of occurrence (Allen 2003; Otto 2017) by making overall conditions more (or less) favorable for extremes (Fischer and Knutti 2015). By evoking both magnitude *and* frequency, studies can compare the occurrence likelihood of an extreme event class (or multiple classes in general attribution) under “factual” conditions (i.e., with anthropogenic influence) with that under “counterfactual” conditions (i.e., with less anthropogenic influence) (Kirchmeier-Young et al. 2017; Swain et al. 2020). Magnitude and frequency are inseparable; both must be evoked to understand if high-magnitude events are increasing in frequency, or if low-frequency events are increasing in magnitude.

## Evaluating two scientific approaches to the forest and floods disagreement

### Evaluating scientific merits and flaws

**Fig. 1:**
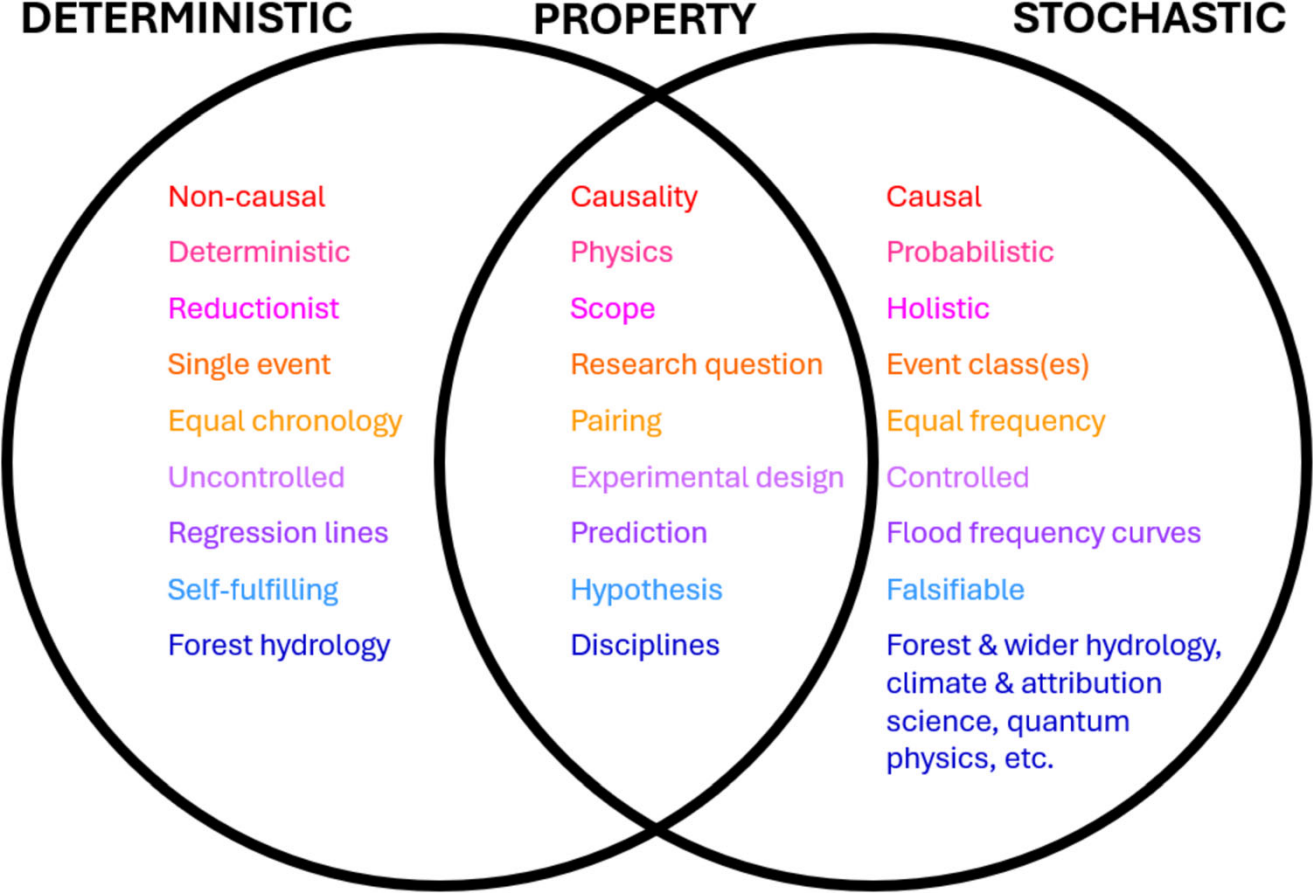
Conceptual diagram showing major differences between the deterministic (left) and stochastic approaches (right) when studying impacts of forest cover changes on flood peaks. Although both utilize the BACI design, differences in ensuing data analysis are shown for various characteristics of the analysis procedure (middle)

While only the stochastic framework captures the *multiple, chancy,* and *dynamically interacting* nature of flood drivers and supports causal claims, critically assessing both deterministic and stochastic approaches allows a deeper understanding of their scientific rigor. By scrutinizing their research questions, hypotheses, and experimental designs (see Fig. 1), this section assesses the suitability of each approach to answer whether forests can mitigate large floods. Although operations like road construction and soil compaction frequently associated with harvesting may influence floods, this review focuses on tree-related effects of forest cover change, such as changes in interception, evapotranspiration, and shading. However, the validity of scientific frameworks and physical reasoning discussed in the following subsections are applicable to investigating non-vegetative changes associated with deforestation.

### Critically assessing the deterministic approach

The deterministic approach is based on the research question, “How does the presence or absence of forest cover alter flood magnitudes for a given rain- or snow-generated event?” (e.g., Bathurst 2014). It is motivated by a perceived need to answer the more general question, “Would that flood have occurred, or would it have been as big, if the forest cover had not been lost?” (Bathurst 2014, p. 2756). Although the latter is argued to be relevant to communities impacted by large floods and answerable only through deterministic methods (e.g., Bathurst et al. 2020), analogous questions are considered “ill-posed” outside of forest hydrology. In their landmark study of the 2003 European heatwave, Stott et al. (2004, p. 610) state that,

It is an ill-posed question whether the 2003 heatwave was caused, in a simple deterministic sense, by a modification of the external influences on climate—for example, increasing concentrations of greenhouse gases in the atmosphere—because almost any such weather event might have occurred by chance in an unmodified climate.

Other prominent climatologists echoed this statement (e.g., Fischer and Knutti 2015), including that science “will never be able to say, at any confidence level, that human influence has contributed x% to an actual weather event” (Allen 2003, p. 892). It is scientifically invalid to deterministically ask whether a given event would have occurred, or by how much it has been exacerbated, due to anthropogenic activity (James et al. 2019). As hydroclimatic extremes result from the dynamic interaction of multiple stochastic variables, understanding human influence requires stochastic framing (Swain et al. 2020; Pham and Alila 2024). In addition to attribution science’s explicit remarks, such questions are hardly asked among causal studies in wider hydrology. With its broader discipline (i.e., hydrology) and sister disciplines (i.e., climate science, attribution science) denouncing the use of such questions to investigate highly analogous topics, forest hydrology’s adherence to them is increasingly scientifically untenable.

The research question is tied to an uncontrolled experimental design that does not isolate the role of changing forest cover on floods, failing to establish the causal link between forest cover and flood generation. Deterministic data analysis compares each rain- or snowmelt-generated peak in the control catchment to the corresponding harvested peak (event-wise chronological pairing), where any differences represent the treatment effect (e.g., Xiao et al. 2022). However, deterministically fixing by one factor fails to account for other factors also recognized to influence flood generation (Alila et al. 2009). For instance, by only accounting for storm size, a *ceteris paribus* conclusion is reached: When all other flood generation factors are held constant, increasing storm size increases flood magnitudes (Pham and Alila 2024). Yet, in rain environments, floods are a function of both rainfall and antecedent catchment wetness, while snowpack accumulation and energy available for snowmelt are additional controls in snow-influenced environments that can be altered by forest removal (Tonina et al. 2008; Ellis et al. 2011; Pham et al. 2025). By considering only one “slice” of flood generation, chronological event-wise pairing does not account for how combinations of flood generation factors differ among flood events and how the whole of flood generation (i.e., all factors and their interactions) is influenced by human activity. Given the presence of unaccounted factors (e.g., antecedent moisture), the effects of changing forest cover on floods are not isolated and the causal link is not established, rendering deterministic conclusions incorrect. It also disregards frequency, which displays much larger changes than magnitude (Allen and Ingram 2002). Therefore, outcomes of the deterministic approach are irrelevant, *and at best misleading*, to whether forests mitigate floods.

**Fig. 2:**
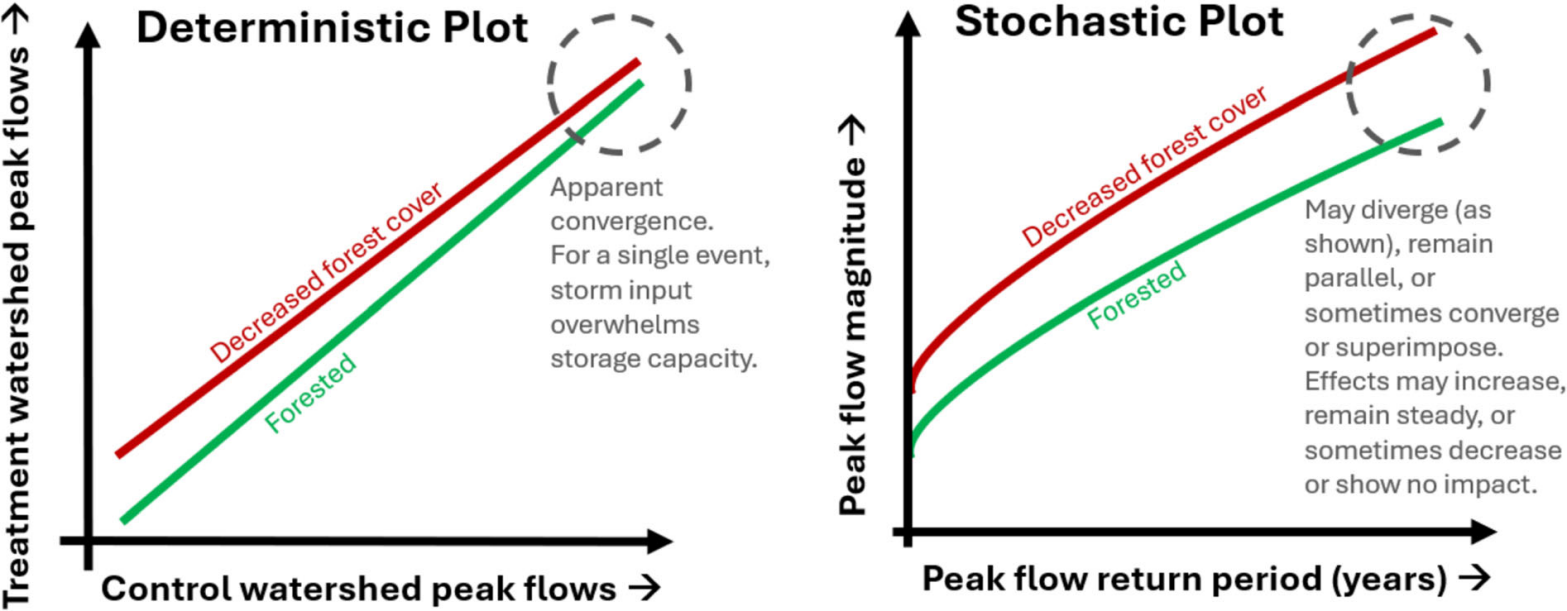
Examples of typical deterministic regression and stochastic flood frequency curve (FFC) plots. While convergence is apparent on deterministic plots, tails may remain parallel, diverge (as shown), converge, or superimpose on stochastic plots

The deterministic hypothesis that forest cover has minimal impact on large flood magnitudes (Bathurst et al. 2011) is also self-fulfilling. While a false hypothesis can narrow down possibilities, untestable theories are “empty” and “misleading” (Kirchner 1989, p. 226). In the latter, “confirmation of the hypothesis does not restrict the sphere of possibilities because the set of excluded data (data that would have been inconsistent with the hypothesis) is empty” (Kirchner 1989, p. 226). Thus, theories become “doomed to inevitable and inconsequential success” (Kirchner 1989, p. 226). Convergence (or apparent convergence) of control and treatment regression lines for the largest event(s) (see Fig. 2) suggests that decreased forest cover primarily increases small- and medium-sized floods with little impact on large events. When a large storm coincides with wet conditions or greatly overwhelms storage capacity, the flood magnitude in the forested catchment must be similar to that in the logged catchment due to little influence of any storage capacity differences on this single event. This must lead to convergence at this peak flow value. In fact, as typical record lengths of a few decades or less (e.g., Harr et al. 1979) only allowed adequate sampling of small and medium events, this deterministic, single-event logic was often extrapolated to large events. While it originated in the rain environment (DeWalle 2003), this logic was also extended to other hydrological regimes including snow (MacDonald and Stednick 2003) and rain-on-snow (Thomas and Megahan 1998; Beschta et al. 2000; Jones 2000).

Yet, even when both watersheds show similar magnitudes for the same large rain- or snowmelt-generated event, there may be other events which are small or medium in the control (which are discarded from the analysis) but are large in the treatment catchment due to other flood generation factors. For example, a small or medium snowmelt event in the forested control may exclude a control–treatment pair, but the corresponding treatment event could be large because decreased forest cover significantly increased the energy available for snowmelt (Alila et al. 2009). In this way, large events could be more frequent in the entire distribution of flood peaks than in a subset of peaks defined by the control catchment. Linkage of frequency to magnitude in stochastic studies (Swain et al. 2020) further suggests that magnitudes change. This directly contrasts deterministic studies, where regression lines must converge (i.e., suggest little effects of forest cover on large floods) for physically meaningful reasons. In fact, Harr (1986, p. 1096) argued that interpreting apparent convergence as evidence of minimal influence of forest cover on large events is “irrelevant” to whether forest removal influences large flood peaks, although this statement has been largely overlooked.

Additionally, deterministic studies paradoxically introduce quantile frequencies (i.e., 5 year, 10 year) when discussing the “threshold” event size beyond which forests have no effects on floods (e.g., Thomas and Megahan 1998; Bathurst et al. 2011). Such patterns may hint at a deep-seated intuition that understanding the flood response requires integrating magnitude *and* frequency and of the key role of frequency in science, management, and policymaking.

In short, the deterministic approach is motivated by an ill-posed question and self-fulfilling hypothesis, leading to a non-causal framework and irrelevant outcome. By failing to causally isolate the effects of changing forest cover on floods, its magnitude conclusions are rendered incorrect and changing frequencies are ignored.

### Critically assessing the stochastic approach

The stochastic approach (e.g., Duncan 1995; Tonina et al. 2008; Te Linde et al. 2010) asks either of two types of probabilistic research questions (i.e., single event or general):

1. For an event of a certain magnitude (frequency), how has a change in forest cover influenced its frequency (magnitude)?
2. How have flood magnitudes and frequencies for small, medium, and large floods changed due to a change in forest cover?

By utilizing magnitude–frequency classes, both questions account for the many combinations by which events of the same magnitude or frequency are generated.

The resulting experimental design is controlled, establishing the causal link between forest cover and flood generation. Instead of event-wise matching, the highest peaks are selected in each watershed (or modeled scenario); then, they are paired by equal magnitude or frequency (e.g., McEachran et al. 2021; Barnes et al. 2023). Peaks are used to construct both pre-disturbance (i.e., forested or less anthropogenically influenced) and post-disturbance (i.e., less forested or more anthropogenically influenced) flood frequency curves (FFCs) to estimate differences in flood magnitudes and frequencies following a disturbance (see Figs. 2 and 4). As FFCs represent magnitude–frequency classes that account for the *multiple, chancy*, and *dynamically interacting* nature of floods, this approach captures the whole of flood generation. It isolates the effects of forest cover changes on floods, answering the ultimate question of interest, “How does the presence or absence of forest cover influence flood magnitudes and frequencies?”

When comparing more forested and less forested FFCs, “upper tails” representing extremes may remain parallel (i.e., forest removal increases or sometimes decreases flood magnitude and frequencies, with effects remaining steady) (e.g., Te Linde et al. 2010), diverge (i.e., increasing effects of forest removal for larger events) (e.g., Reynard et al. 2001), converge (i.e., decreasing effects of forest removal for larger events) (e.g., Green and Alila 2012), or sometimes superimpose on each other (i.e., no effects) (see Fig. 2). As all are possibilities (see later discussion in this subsection), the hypothesis that forest cover can decrease both magnitudes and frequencies for large flood events is falsifiable.

**Fig. 3:**
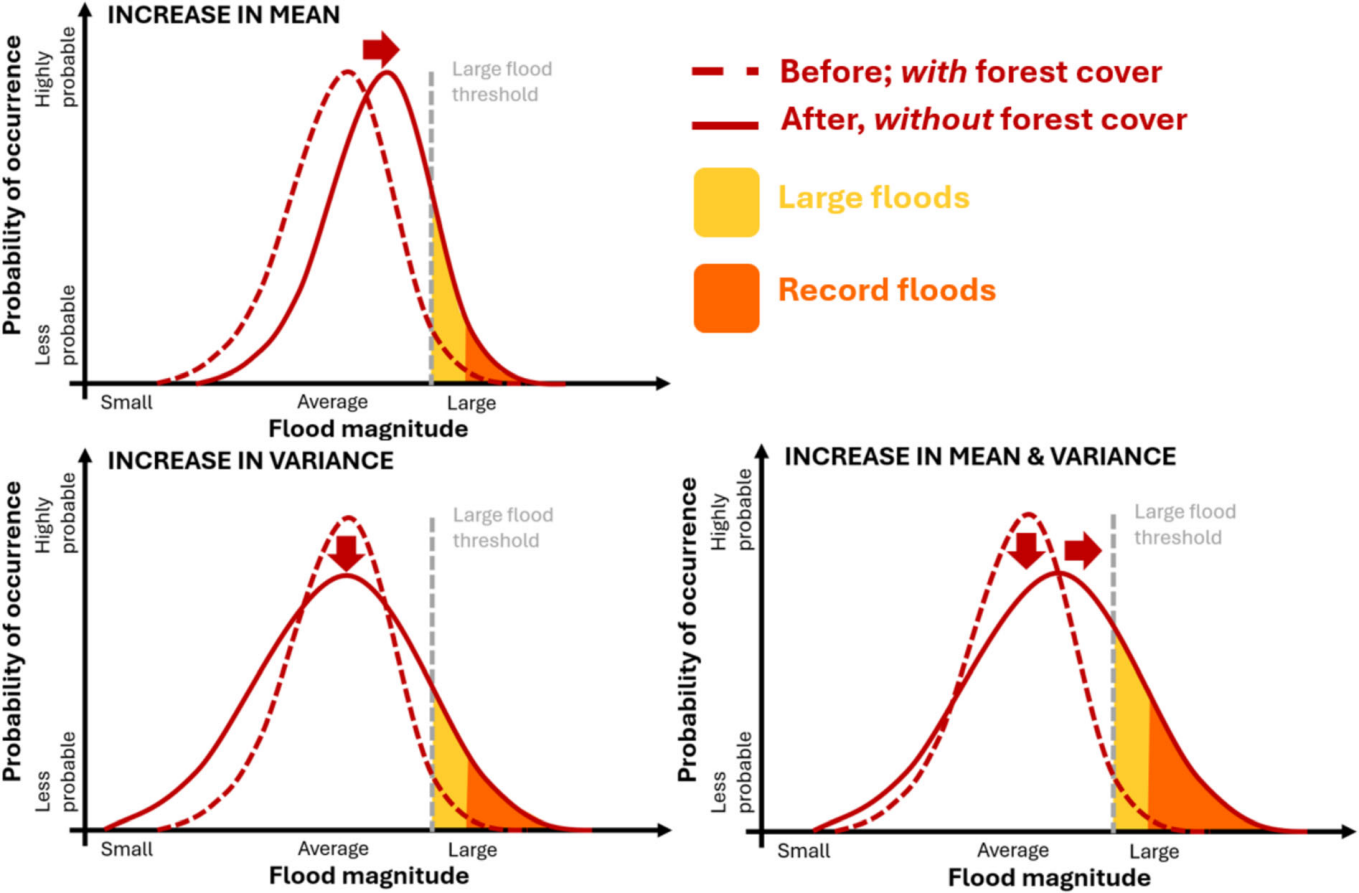
Conceptual changes from an increase in mean, variance, or both mean and variance to a hypothetical flood probability distribution. The gray dashed line represents a hypothetical threshold above which floods are considered large, with increases in mean, variance, or both mean and variance increasing the number of large and record floods. While anthropogenic activity can also change the shape (skewness) of the distribution, it is not depicted here for simplicity

Incorporating sound physical insight is also central to stochastic hydrology, allowing scientifically defensible predictions that better reflect hydrologic reality. Both climate and physiography control the “natural” FFC, with climatic controls prevailing over physiographic controls after storage capacity is overwhelmed (Macdonald et al. 2024). Notably, the GRADEX (GRADient of Extreme values) concept suggests that flood tails parallel those of rainfall after catchment storage is exceeded, with the vertical difference between the two tails representing stored precipitation within the catchment (Naghettini et al. 2012; Pham and Alila 2024). Natural characteristics control the shape of the FFC, leading to a “lighter” (i.e., goes to zero faster than the exponential) or “heavier” (i.e., goes to zero slower than the exponential) tail (Kaluarachchi and Alila 2025) with implications for understanding watershed sensitivity and flood management (see Section "Revisiting the scientific disagreement"). Anthropogenic activity such as forest removal or climate change acts upon these influences, shifting the natural FFC to its post-disturbance position. Changes in the FFC reflect how mean, variance, and skewness of flood populations change (see Fig. 3); while changes in the mean influence frequencies of extremes (Mearns et al. 1984), changes in variability can be much more consequential (Katz and Brown 1992), leading to divergence (i.e., increased variability) or convergence (i.e., decreased variability) of the FFCs (Green and Alila 2012). By tying these changes to physical causes, stochastic hydrology supports a physically based understanding of change.

**Fig. 4:**
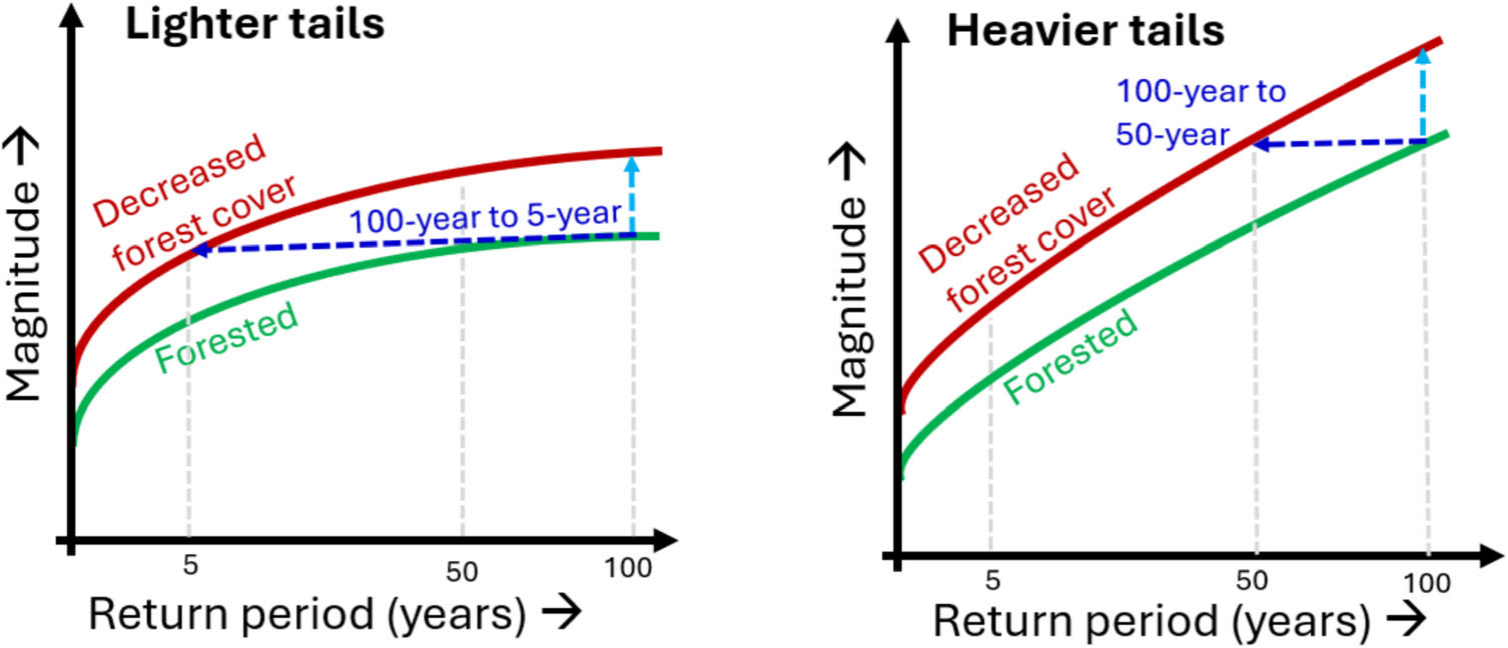
Hypothetical changes in magnitude (light blue arrow) and frequency (dark blue arrow) with a decrease in forest cover under both lighter- and heavier-tailed flood regimes, adapted from Kaluarachchi and Alila (2025)

Decreased forest cover typically leads to an “upward” shift of the FFC relative to the more forested FFC (see Fig. 4), with divergence (e.g., Reynard et al. 2001; Pham et al. 2025), parallelism (e.g., Tonina et al. 2008; Hurkmans et al. 2009; Te Linde et al. 2010; Lallemant et al. 2021), or convergence (e.g., Green and Alila 2012) (or in some cases, little or reversed effects (e.g., Pham et al. 2025)) at the tails, all depending on climatic and physiographic features. Increased evapotranspiration and moisture loss under forest cover (Duncan 1995) can decrease catchment water storage. This translates to an increase in available storage capacity and greater attenuation of runoff, consistent with the general hydrological understanding that any increase in storage capacity should increase attenuation of runoff (e.g., Macdonald et al. 2024). Conversely, wetter soils under less forested conditions can reduce available storage capacity and decrease potential for attenuating runoff, favoring more frequent and higher-magnitude floods (Duncan 1995). In snow environments, greater snow accumulation increases moisture availability for flood generation and greater energy input causes faster snowmelt (Green and Alila 2012). However, it is catchment scale characteristics such as cut rates, locations of cutblocks, and physiographical variations which govern the overall flood response and effects on the FFC. For instance, greater heterogeneity in slope aspects and elevations and appropriate harvesting schemes can support “time-staggered” conveyance of snowmelt-generated flows to the outlet, from lower to higher elevations and from south-facing to north-facing aspects in the northern hemisphere, dampening effects on large events and leading to convergence (Ellis et al. 2011; Green and Alila 2012; Johnson and Alila 2023). Low physiographic heterogeneity may promote simultaneous melt and similar timings at which flows reach the outlet, suggesting larger effects on large flood events (i.e., divergence) (Green and Alila 2012; Johnson and Alila 2023). This stochastic understanding explains why FFCs generally shift “up” (“down”) following a decrease (an increase) in forest cover and why effects may increase or decrease for extreme events.

The stochastic interpretation is fundamentally different from the deterministic interpretation which focuses on a single event (i.e., when precipitation exceeds available storage capacity, forest cover has little impact on the event). The stochastic interpretation integrates space and time (Robinson and Sivapalan 1997), focusing on classes of events. While large floods can still occur (Barnes et al. 2023), forest cover makes overall conditions less favorable for large floods, leading to an overall decrease in flood magnitudes and (especially) frequencies. As emphasized by attribution science, even small changes in magnitude lead to disproportionately large changes in frequency (see Fig. 4) (Allen and Ingram 2002; Lloyd and Shepherd 2020). These large frequency changes are masked by the deterministic approach, but emerge under stochastic approaches.

In conclusion, results of the stochastic approach are clear: Forest cover can attenuate floods of all sizes, even when deterministic methods suggest little influence of forest cover on the largest floods (e.g., Birkinshaw et al. 2011; Green and Alila 2012).

## Revisiting the role of forests in flood management

### Revisiting the scientific disagreement

**Fig. 5:**
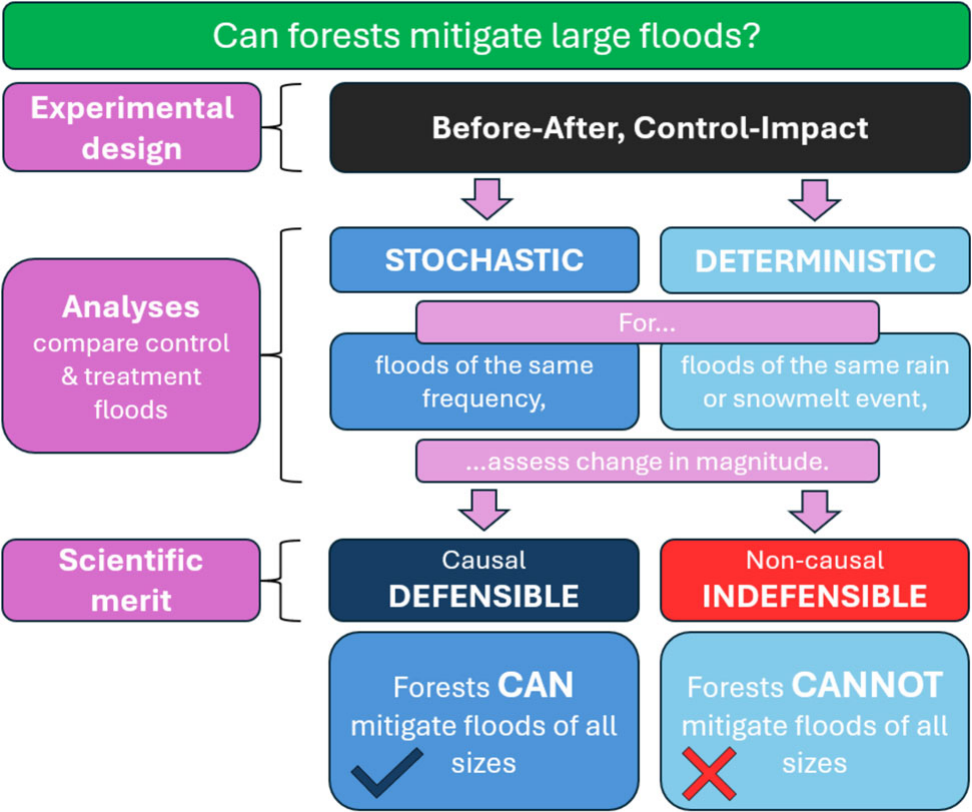
Synthesis of the stochastic and deterministic approaches to data analysis following the Before-After, Control-Impact (BACI) approach to answer whether forests mitigate large floods. The analyses, typical conclusions, and scientific rigor described here also apply to modeled catchments with differing land cover scenarios

Scientific skepticism toward forest-based NBS stems from the deterministic understanding that forests have little impact following catchment saturation (Dadson et al. 2017; Barnes et al. 2023). However, its irrelevant research question, self-fulfilling hypothesis, and uncontrolled design indicate low scientific defensibility of its conclusions (see Fig. 5). The stochastic approach instead suggests that forests can mitigate floods of all sizes (e.g., Reynard et al. 2001; Hurkmans et al. 2009; Te Linde et al. 2010) using strong research questions, falsifiable hypotheses, and a controlled experimental design for scientifically rigorous conclusions. Stochastic studies are thus encouraged within forest hydrology (e.g., Tonina et al. 2008; Birkinshaw 2014), wider hydrology (Brath et al. 2006; Brunner et al. 2021), and climatology and attribution science (e.g., Otto 2017) to investigate anthropogenic impacts on floods (e.g., Gillett et al. 2022) and other extremes (e.g., Kirchmeier-Young et al. 2017; Stott et al. 2004). Stark asymmetry in scientific defensibility and contradictory conclusions suggests that the scientifically indefensible approach must be replaced by the rigorous approach.

While some suggest that both approaches can co-exist (Bathurst 2014; Birkinshaw 2014), science cannot simultaneously maintain two contradictory sets of conclusions (i.e., increasing forest cover both mitigates and does not mitigate large floods). The causal framework widely recognized in multiple disciplines must be upheld. Furthermore, attempts to maintain both approaches often suggest that frequency changes are independent of magnitude changes, allowing event frequencies to increase, while magnitudes do not (Dadson et al. 2017; Barnes et al. 2023). However, magnitude and frequency are both needed to causally understand and predict flood extremes. Causal inference must be informed by the full distribution of peak flows, rather than isolated events or subsets defined by flood size or flood generation factors that remove them from a common magnitude–frequency framework (Alila et al. 2009). This may explain why the few regression-based studies that preserve this flood frequency distribution rather than relying on event-based analyses tend to show negative associations between forest cover and flood frequency (e.g., Bradshaw et al. 2007; Huang et al. 2018), consistent with stochastic results (albeit non-causal). Furthermore, magnitude and frequency are two sides of the same coin (Swain et al. 2020); thus, a change in frequency necessitates and is accompanied by a change in magnitude (and vice versa) in the causal framework. For instance, if a former 100-year flood is now the 25-year event, the magnitude corresponding to the former 100-year event now corresponds to that of the 25-year event; thus, the new 100-year magnitude is larger. Accepting stochastic frequency conclusions *necessitates* accepting stochastic magnitude conclusions for a rigorous, scientifically defensible understanding.

This may lead to the question of whether both approaches could co-exist if they produce the same answer (e.g., Liu et al. 2025). Contradictory conclusions between the two bodies of literature demonstrate that they rarely produce the same answer. Furthermore, convergence of deterministic regression lines does not equate to the convergence of FFCs. In the former, convergence must occur; thus, it does not exclude the possibility that forest cover mitigates large floods. In contrast, FFCs may diverge, remain parallel, or sometimes converge or superimpose.

Most importantly, as society enters unchartered territories in the Anthropocene, only a sound, causal understanding can guide flood projections and adaptation (Kaluarachchi and Alila 2025). Regardless of how answers compare, science must ensure it is “getting the right answers for the right reasons” (Kirchner 2006, p. 1).

## Research directions, management, and policy

Causal, probabilistic results must motivate strong policy, planning, industry practices, and flood management. While attribution studies of the role of climate change on floods have received much attention, they typically do not examine other drivers or the vulnerability and exposure of populations to risk (James et al. 2019; Lahsen and Ribot 2022). This is despite many studies causally linking deforestation to increased flooding (including 18-fold increases in flood frequency (Pham et al. 2025)) as well as the public and media questioning the roles of both climate change and deforestation in exacerbating recent floods (e.g., Gilliver 2025). A study which mapped over 1300 geohazards triggered by the 2021 flooding in southwestern British Columbia (BC) found that roughly half of them originated in areas with disturbed forest cover, including from logging cutblocks (15%), forest resource roads (14%), and wildfire (17%), suggesting associations between forest disturbance and geohazards (Hancock and Wlodarczyk 2025). These examples highlight a need for causal attribution of changing flood risks (whether single event or general) to relevant climatic *and* land use drivers, all of which can inform exposure, vulnerability, and other impacts.

Studies identifying or attributing change must account for possible nonstationarities in data (i.e., changes in statistical properties of flood distributions over time) induced by LULC changes. For example, although the Water Survey of Canada (WSC) selects reference basins with minimal human influence to support long-term trend analysis, basin selection does not account for forest cover change, yet many studies using WSC data assume stable land cover (Leach et al. 2025). Basin selection and flood studies must no longer assume stationarity in areas with past or present LULC change. Moreover, using non-causal methods for trend analysis can lead to difficulty and ambiguity in distinguishing climatic versus LULC causes of change (e.g., Leach et al. 2025), increasing the potential for misidentifying climate change as the sole driver of change when changes in forest cover could play a notable role. In contrast, use of causal approaches incorporating modeling and/or nonstationary frameworks (Brunner et al. 2021; McEachran et al. 2021) allows for rigorous consideration of nonstationarity and attribution of change.

Furthermore, as typical record lengths are insufficient for capturing large events (e.g., Merz and Blöschl 2009), larger sample sizes can be simulated to complement observational records (Brunner et al. 2021). Even where sample sizes are limited, sound hydrological reasoning (Merz and Blöschl 2008), including physically based interpretation of change, increases the reliability of flood predictions. For instance, applying the GRADEX logic to changes in forest cover suggests that decreased available storage capacity following deforestation must lead to an earlier return period at which flood tails mimic that of rainfall (i.e., deforested tail is shifted “up” relative to the forested tail), with the vertical difference between the tails representing differences in storage capacity between the two scenarios (Pham and Alila 2024).

In attribution studies, although magnitude changes must be evoked and considered, larger frequency changes are highly impactful for planning and decision making (Swain et al. 2020). For example, while smaller increases in magnitude may appear inconsequential to the public and policymakers, a former 100-year event becoming a 25-year event can motivate action. Large frequency increases can also be detrimental for aquatic and riparian species (Poff et al. 1997) and increase potential for geomorphic failures (Flint et al. 2017); thus, both frequency *and* magnitude must be considered in impact assessments.

As most work regarding the effects of changing forest cover on floods is deterministic, stochastic studies evaluating the effects of forest disturbance and afforestation/reforestation on (large) floods are needed, especially in a changing climate. Studies should be carried out under various hydroclimatic regimes, physiographies, vegetation types and densities, and harvesting characteristics (e.g., cutblock locations, clearcut versus selective logging, thinning (Chausson et al. 2020)). Moreover, high implementation costs remain a major barrier to the adoption of NBS (Jongman 2018; Seddon et al. 2020) despite their long-term cost-effectiveness (Faivre et al. 2018). Stochastic studies can thus strengthen the case for funding and identify which interventions or designs best mitigate large floods for increased cost-effectiveness. They can also provide clearer, causal, and more consistent evidence to policymakers on the role of forests in flood mitigation (Bradshaw et al. 2009), helping inform effective policy. Governmental and technical guidance reflecting non-causal conclusions (e.g., Stratford et al. 2017; Environment Agency 2018) should in turn be updated to reflect causal science.

Some flood regimes are naturally highly sensitive to anthropogenic disturbance, requiring careful management of land use and economic activities to avoid escalating flood risk. The tail behavior and controls of the natural FFC indicate sensitivity; lighter-tailed FFCs are highly sensitive and can thus yield large changes in flood frequencies following forest removal (see Fig. 4) (Kaluarachchi and Alila 2025). An example is BC, where characteristics such as the influence of snow (Merz and Blöschl 2003) and frontal storms (Pitlick 1994), environmental heterogeneity (Green and Alila 2012), and large catchment areas (Yang et al. 2019) promote lighter tails in many regions (e.g., Loukas et al. 2000; Beckers et al. 2002). Disturbing these landscapes could thus produce substantial changes to flood regimes. In fact, stochastic studies have demonstrated large increases in flood frequencies following harvesting, including a tenfold increase in the 100-year event in Interior BC (Johnson and Alila 2023). Identifying sensitive watersheds using physically informed flood frequency (stochastic) analysis (Kaluarachchi and Alila 2025) can guide sustainable harvesting (e.g., choosing less sensitive locations, harvesting in ways that desynchronize snowmelt) and nature-based flood management.

Flood management should aim for holistic, landscape-level planning (e.g., Chausson et al. 2020) that integrates nature-based and traditional solutions, with NBS given priority for their long-term benefits to society and ecosystems (Peck et al. 2022). Stochastic studies suggest that maintaining or increasing forest cover can greatly reduce flood risk at the source, particularly in sensitive landscapes like large, lighter-tailed catchments in BC (Johnson and Alila 2023) and in regions facing increasing climate-driven flood risk (Reynard et al. 2001). They can also reduce economic losses and populations exposed to risk, even for 500-year events (Lallemant et al. 2021). Reforestation or afforestation efforts should prioritize appropriate (e.g., native) species, promote diverse plant communities over monocultures, support biodiversity, and enhance habitat connectivity to facilitate the movement of species in response to stressors (Seddon et al. 2021). Implications of forest cover and greater storage capacity can also be used to design downstream flood management strategies such as urban forests, green spaces in cities, and detention basins. In this way, planning, management, and policy all hinge on a sound, causal understanding of flood risk and its multiple drivers.

## Conclusion

Rising flood frequencies and magnitudes worldwide are leading to severe socioeconomic impacts, underscoring the urgent need for effective flood management. While nature-based flood management has gained traction for its multiple benefits, it is at the center of a long-standing scientific controversy over the role of forest cover in mitigating (large) floods. Decades of forest hydrology research continue to maintain two contradictory sets of conclusions (i.e., forests do and do not mitigate large floods) with controversy more amplified now than ever before, impairing flood prediction, policymaking, and solution design at a time when strong management is critical. This demands a closer examination of whether all conclusions are equally valid or whether one rests on more defensible scientific ground. Compared to the non-causal deterministic method, only the causal, stochastic approach captures the probabilistic nature of flood generation and incorporates a relevant research question, falsifiable hypothesis, controlled experimental design, and sound physical reasoning. Its scientific rigor and suitability, especially for studying stochastic phenomena, have led to its widespread acceptance throughout hydroclimatology and other sciences. Yet, much of the discourse in forest hydrology has focused on reconciling these fundamentally irreconcilable methods, perhaps reflecting fear of invalidating a century of deterministic research or a political interest in maintaining methods which largely suggest little influence of harvesting on large floods. However, reliance on non-relevant and non-causal methods poses real consequences; they increase flood risk, put communities in harm’s way, and can strain public budgets by funding ineffective solutions or from damages associated with extreme floods. In contrast, a causal, stochastic understanding informs planning and policymaking, cost-effective flood management, sustainable resource management, and adaptation. With rigorous, defensible knowledge, science can help develop strategies which meaningfully reduce flood risk and are practical for local, regional, and national governments.

## Supplementary Information

Below is the link to the electronic supplementary material.

Supplementary file1 (PDF 957 KB)
