# Supplementary material for: Why forests can mitigate floods of all sizes: Evaluating the scientific basis for forest-based flood mitigation
Source: Ambio. 2026 Feb 1;55(7):1478–92. doi: 10.1007/s13280-026-02346-6 (PMC13230409; doi:10.1007/s13280-026-02346-6)
Supplement: Supplementary file 1 — Supplementary file1 (PDF 957 KB) [file 13280_2026_2346_MOESM1_ESM.pdf]

Supplementary Information: This Supplementary Information has not been peer reviewed.

Title: Why forests can mitigate floods of all sizes: Evaluating the scientific basis for forest-based flood mitigation

Table S1. Overview of studies quantitatively investigating the effects of forest cover changes on peak flows using a deterministic and/or stochastic analysis procedure following a Before-After, Control-Impact (BACI) or modelled catchment design. Review studies without quantitative data analysis and studies investigating effects to other variables such as water yield without examining peak flows are not included. For each cited study, the following information is provided: location(s) with any applicable provinces or states in parentheses, catchment size(s) including both control and treatment catchments, hydroclimatic regime(s), whether it is a field/paired or modelled catchment study, and whether it is deterministic and/or stochastic.

**Citation**	**Location(s)**	**Catchment Size(s) (km²)**	**Hydroclimatic Regime(s) (ROS = rain-on-snow)**	**Field/Paired (F/P) or Modelled (M)**	**Deterministic (D) or Stochastic (S)**
(Alila et al., 2009)	United States (Colorado, Oregon)	0.6–8.03	Snow & ROS	F/P	D & S
(Barnes et al., 2023)	United Kingdom	335 (headwaters: 1–10)	Rain	M	D & S
(Bathurst et al., 2011)	Ecuador, Chile, Costa Rica, Argentina	0.6–1545	Rain & ROS	F/P	D
(Bathurst et al., 2018)	United Kingdom	1.4–6.9	Rain	F/P	D & S

(Bathurst et al., 2020)	Chile, New Zealand, United Kingdom, United States (Oregon)	0.34–3.1	Primarily rain	F/P	D & S
(Beschta et al., 2000)	United States (Oregon)	0.6–640	ROS	F/P	D
(Birkinshaw et al., 2011)	Chile	0.35	Rain	M	D & S
(Birkinshaw et al., 2014)	United Kingdom	1.5	Primarily rain	M	D & S
(Burton, 1997)	United States (Utah)	21.34–22.33	Snow	F/P	D
(Cheng, 1989)	Canada (British Columbia)	33.9–40.7	Snow	F/P	D
(Duncan, 1995)	New Zealand	0.04-0.077	Rain	F/P	S
(Fahey & Payne, 2017)	New Zealand	2.16-3.1	Primarily rain	F/P	D & S
(Goodbrand et al., 2022)	Canada (Alberta)	15.2–28.3	Rain & snow	F/P	D
(Green & Alila, 2012)	Canada (British Columbia),	3–37	Snow	F/P & M	D & S

	United States (Colorado)				
(Guillemette et al., 2005)	Canada (Quebec)	1.22–3.94	Rain	F/P	D
(Harr et al., 1979)	United States (Oregon)	0.486–0.692	Rain & snow	F/P	D
(Harr & McCorison, 1979)	United States (Oregon)	0.085–0.102	Rain, snow, & ROS	F/P	D
(Harr, 1980)	Oregon (United States)	0.59–2.53	Rain, snow, & ROS	F/P	D
(Harr et al., 1982)	United States (Oregon)	0.13–21.4	Snow & ROS	F/P	D
(Harr, 1986)	United States (Oregon)	0.085–0.96	ROS	F/P	D
(Harris, 1977)	United States (Oregon)	0.70–3.03	Rain	F/P	D
(Hewlett & Helvey, 1970)	Unites States (North Carolina)	0.44–0.49	Rain	F/P	D
(Hornbeck et al., 1970)	United States (New England)	0.16–0.42	Rain & snow	F/P	D
(Hornbeck, 1973)	United States (New Hampshire)	0.156–0.424	Rain & snow	F/P	D

(Hurkmans et al., 2009)	Rhine Basin in west Europe	185000 (tributaries: 4118–27088)	Rain & snow	M	S
(Johnson & Alila, 2023)	Canada (British Columbia)	99–1011	Snow	F/P	S
(Jones & Grant, 1996)	United States (Oregon)	0.6–600	Rain & ROS	F/P	D
(Jones, 2000)	United States (Oregon)	0.1–2.53	Rain & ROS	F/P	D
(King, 1989)	United States (Idaho)	0.22–1.48	Snow & ROS	F/P	D
(Kuraś et al., 2012)	Canada (British Columbia)	4.74	Snow	M	S
(Lallemant et al., 2021)	Myanmar	113800	Rain	M	S
(McEachran et al., 2021)	United States (Minnesota)	0.089–0.526	Rain, snow, & ROS	F/P	D & S
(Moore & Scott, 2005)	Canada (British Columbia)	33.9–40.7	Snow	F/P	D
(Pham et al., 2025)	United States (North Carolina)	0.12–0.16	Rain	F/P	S
(Reynard et al., 2001)	United Kingdom	9895–9948	Rain	M	S

(Rothacher, 1973)	United States (Oregon)	0.61–0.96	ROS	F/P	D
(Schnorbus & Alila, 2004)	Canada (British Columbia)	26	Snow	M	S
(Schnorbus & Alila, 2013)	Canada (British Columbia)	4.70	Snow	M	S
(Storck et al., 1998)	United States (Washington)	5.2–388	Snow & ROS	M	D
(Te Linde et al., 2010)	Rhine Basin in northwest Europe	185000	Rain & snow	M	S
(Thomas & Megahan, 1998)	United States (Oregon)	0.6–600	Rain & ROS	F/P	D
(Tonina et al., 2008)	United States (Idaho)	12.6–30.2	Snow & ROS	M	S
(Troendle & King, 1985)	United States (Colorado)	2.89–8.03	Snow	F/P	D
(Troendle & King, 1987)	United States (Colorado)	0.41–7.67	Snow	F/P	D
(Troendle et al., 2001)	United States (Wyoming)	9.08–16.73	Snow	F/P	D

(Ursic, 1991)	United States (Mississippi)	0.0134–0.0185	Rain	F/P	D
(Van Haveren, 1988)	United States (Colorado)	0.81–0.9	Snow	F/P	D
(Verry et al., 1983)	United States (Minnesota)	0.34–0.53	Rain & snow	F/P	D & S
(Walsh et al., 2020)	Australia (New South Wales)	0.013–0.048	Rain	F/P	D
(Xiao et al., 2022)	United Kingdom, Ireland	0.08–3.9	Rain	F/P	D

References

Alila, Y., Kuraś, P. K., Schnorbus, M., & Hudson, R. (2009). Forests and floods: A new paradigm sheds light on age-old controversies. *Water Resources Research*, *45*(8). https://doi.org/10.1029/2008WR007207

Barnes, M. S., Bathurst, J. C., Lewis, E., & Quinn, P. F. (2023). Leaky dams augment afforestation to mitigate catchment scale flooding. *Hydrological Processes*, *37*(6). https://doi.org/10.1002/hyp.14920

Bathurst, J. C., Birkinshaw, S., Johnson, H., Kenny, A., Napier, A., Raven, S., Robinson, J., & Stroud, R. (2018). Runoff, flood peaks and proportional response in a combined nested and paired forest plantation/peat grassland catchment. *Journal of Hydrology*, *564*, 916–927. https://doi.org/10.1016/j.jhydrol.2018.07.039

Bathurst, J. C., Fahey, B., Iroumé, A., & Jones, J. (2020). Forests and floods: Using field evidence to reconcile analysis methods. *Hydrological Processes*, *34*(15), 3295–3310. https://doi.org/10.1002/hyp.13802

Bathurst, J. C., Iroumé, A., Cisneros, F., Fallas, J., Iturraspe, R., Novillo, M. G., Urciuolo, A., Bièvre, B. de, Borges, V. G., Coello, C., Cisneros, P., Gayoso, J., Miranda, M., & Ramírez, M. (2011). Forest impact on floods due to extreme rainfall and snowmelt in four Latin American environments 1: Field data analysis. *Journal of Hydrology*, *400*(3–4), 281–291. https://doi.org/10.1016/j.jhydrol.2010.11.044

Beschta, R. L., Pyles, M. R., Skaugset, A. E., & Surfleet, C. G. (2000). Peakflow responses to forest practices in the western cascades of Oregon, USA. *Journal of Hydrology*, *233*, 102–120. www.elsevier.com/locate/jhydrol

Birkinshaw, S. J., Bathurst, J. C., Iroumé, A., & Palacios, H. (2011). The effect of forest cover on peak flow and sediment discharge-an integrated field and modelling study in central-southern Chile. *Hydrological Processes*, *25*(8), 1284–1297. https://doi.org/10.1002/hyp.7900

Birkinshaw, S. J., Bathurst, J. C., & Robinson, M. (2014). 45 years of non-stationary hydrology over a forest plantation growth cycle, Coalburn catchment, Northern England. *Journal of Hydrology*, *519*(PA), 559–573. https://doi.org/10.1016/j.jhydrol.2014.07.050

Burton, T. A. (1997). Effects of basin-scale timber harvest on water yield and peak streamflow. *Journal of the American Water Resources Association*, *33*(6), 1187–1196. https://doi.org/10.1111/j.1752-1688.1997.tb03545.x

Cheng, J. D. (1989). Streamflow changes after clear-cut logging of a pine beetle-infested watershed in southern British Columbia, Canada. *Water Resources Research*, *25*(3), 449–456. https://doi.org/10.1029/WR025i003p00449

Duncan, M. J. (1995). Hydrological impacts of converting pasture and gorse to pine plantation, and forest harvesting, Nelson, New Zealand. *Journal of Hydrology (New Zealand)*, *34*(1).

Fahey, B., & Payne, J. (2017). The Glendhu experimental catchment study, upland east Otago, New Zealand: 34 years of hydrological observations on the afforestation of tussock grasslands. *Hydrological Processes*, *31*(16), 2921–2934. https://doi.org/10.1002/hyp.11234

Goodbrand, A., Anderson, A., Devito, K., & Silins, U. (2022). Untangling harvest-streamflow responses in foothills conifer forests: Nexus of teleconnections, summer-dominated precipitation, and storage. *Hydrological Processes*, *36*(2). https://doi.org/10.1002/hyp.14479

Green, K. C., & Alila, Y. (2012). A paradigm shift in understanding and quantifying the effects of forest harvesting on floods in snow environments. *Water Resources Research*, *48*(10). https://doi.org/10.1029/2012WR012449

Guillemette, F., Plamondon, A. P., Prévost, M., & Lévesque, D. (2005). Rainfall generated stormflow response to clearcutting a boreal forest: Peak flow comparison with 50 world-wide basin studies. *Journal of Hydrology*, *302*(1–4), 137–153. https://doi.org/10.1016/j.jhydrol.2004.06.043

Harr, R. D. (1980). *Streamflow After Patch Logging in Small Drainages Within the Bull Run Municipal Watershed, Oregon*.

Harr, R. D. (1986). Effects of Clearcutting on Rain-on-Snow Runoff in Western Oregon: A New Look at Old Studies. *Water Resources Research*, *22*(7), 1095–1100. https://doi.org/10.1029/WR022i007p01095

Harr, R. D., Fredriksen, R. L., & Rothacher, J. (1979). Changes in streamflow following timber harvest in southwestern Oregon. *USDA Forest Service, Research Papers*, *PNW 249*.

Harr, R. D., Levno, A., & Mersereau, R. (1982). Streamflow changes after logging 130-year-old Douglas fir in two small watersheds. *Water Resources Research*, *18*(3), 637–644. https://doi.org/10.1029/WR018i003p00637

Harr, R. D., & McCorison, F. M. (1979). Initial effects of clearcut logging on size and timing of peak flows in a small watershed in western Oregon. *Water Resources Research*, *15*(1), 90–94. https://doi.org/10.1029/WR015i001p00090

Harris, D. D. (1977). Hydrologic changes after logging in two small Oregon coastal watersheds. *Geological Survey Water-Supply Paper 2037*.

Hewlett, J. D., & Helvey, J. D. (1970). Effects of Forest Clear-Felling on the Storm Hydrograph. *Water Resources Research*, *6*(3), 768–782. https://doi.org/10.1029/WR006i003p00768

Hornbeck, J. W. (1973). Storm flow from hardwood-forested and cleared watersheds in New Hampshire. *Water Resources Research*, *9*(2), 346–354. https://doi.org/10.1029/WR009i002p00346

Hornbeck, J. W., Pierce, R. S., & Federer, C. A. (1970). Streamflow Changes after Forest Clearing in New England. *Water Resources Research*, *6*(4), 1124–1132. https://doi.org/10.1029/WR006i004p01124

Hurkmans, R. T. W. L., Terink, W., Uijlenhoet, R., Moors, E. J., Troch, P. A., & Verburg, P. H. (2009). Effects of land use changes on streamflow generation in the Rhine basin. *Water Resources Research*, *45*(6). https://doi.org/10.1029/2008WR007574

Johnson, R. S. H., & Alila, Y. (2023). Nonstationary stochastic paired watershed approach: Investigating forest harvesting effects on floods in two large, nested, and snow-dominated watersheds in British Columbia, Canada. *Journal of Hydrology*, *625*. https://doi.org/10.1016/j.jhydrol.2023.129970

Jones, J. A. (2000). Hydrologic processes and peak discharge response to forest removal, regrowth, and roads in 10 small, experimental basins, western Cascades, Oregon. *Water Resources Research*, *36*(9), 2621–2642. https://doi.org/10.1029/2000WR900105

Jones, J. A., & Grant, G. E. (1996). Peak flow responses to clear-cutting and roads in small and large basins, western Cascades, Oregon. *Water Resources Research*, *32*(4), 959–974. https://doi.org/10.1029/95WR03493

King, J. G. (1989). Streamflow responses to road building and harvesting: a comparison with the equivalent clearcut area procedure. *Research Paper - US Department of Agriculture, Forest Service*, *INT-401*.

Kuraś, P. K., Alila, Y., & Weiler, M. (2012). Forest harvesting effects on the magnitude and frequency of peak flows can increase with return period. *Water Resources Research*, *48*(1). https://doi.org/10.1029/2011WR010705

Lallemant, D., Hamel, P., Balbi, M., Lim, T. N., Schmitt, R., & Win, S. (2021). Nature-based solutions for flood risk reduction: A probabilistic modeling framework. *One Earth*, *4*(9), 1310–1321. https://doi.org/10.1016/j.oneear.2021.08.010

McEachran, Z. P., Karwan, D. L., Sebestyen, S. D., Slesak, R. A., & Ng, G. H. C. (2021). Nonstationary flood-frequency analysis to assess effects of harvest and cover type conversion on peak flows at the Marcell Experimental Forest, Minnesota, USA. *Journal of Hydrology*, *596*. https://doi.org/10.1016/j.jhydrol.2021.126054

Moore, R. D., & Scott, D. F. (2005). Camp Creek Revisited: Streamflow Changes Following Salvage Harvesting in a Medium-Sized, Snowmelt-Dominated Catchment. *Canadian Water Resources Journal*, *30*(4), 331–344. https://doi.org/10.4296/cwrj3004331

Pham, H. C., Alila, Y., & Caldwell, P. V. (2025). Stochastic framework reveals the controls of forest treatment – peakflow causal relations in rain environment. *Journal of Hydrology*, *661*, 133704. https://doi.org/10.1016/J.JHYDROL.2025.133704

Reynard, N. S., Prudhomme, C., & Crooks, S. M. (2001). *The flood characteristics of large U.K. rivers: Potential effects of changing climate and land use*.

Rothacher, J. (1973). *Does harvest in west slope Douglas-fir increase peak flow in small forest streams?*

Schnorbus, M., & Alila, Y. (2004). Forest harvesting impacts on the peak flow regime in the Columbia Mountains of southeastern British Columbia: An investigation using long-term numerical modeling. *Water Resources Research*, *40*(5). https://doi.org/10.1029/2003WR002918

Schnorbus, M., & Alila, Y. (2013). Peak flow regime changes following forest harvesting in a snow-dominated basin : Effects of harvest area, elevation, and channel connectivity. *Water Resources Research*, *49*(1), 517–535. https://doi.org/10.1029/2012WR011901

Storck, P., Bowling, L., Wetherbee, P., & Lettenmaier, D. (1998). Application of a GIS-based distributed hydrology model for prediction of forest harvest effects on peak stream flow in the Pacific Northwest. *Hydrological Processes*, *12*(6), 889–904. https://doi.org/10.1002/(SICI)1099-1085(199805)12:6<889::AID-HYP661>3.0.CO;2-P

Te Linde, A. H., Aerts, J. C. J. H., & Kwadijk, J. C. J. (2010). Effectiveness of flood management measures on peak discharges in the Rhine basin under climate change. *Journal of Flood Risk Management*, *3*(4), 248–269. https://doi.org/10.1111/j.1753-318X.2010.01076.x

Thomas, R. B., & Megahan, W. F. (1998). Peak flow responses to clear-cutting and roads in small and large basins, western Cascades, Oregon: A second opinion. *Water Resources Research*, *34*(12), 3393–3403. https://doi.org/10.1029/98WR02500

Tonina, D., Luce, C. H., Rieman, B., Buffington, J. M., Goodwin, P., Clayton, S. R., Alì, S. M., Barry, J. J., & Berenbrock, C. (2008). Hydrological response to timber harvest in northern Idaho: Implications for channel scour and persistence of salmonids. *Hydrological Processes*, *22*(17), 3223–3235. https://doi.org/10.1002/hyp.6918

Troendle, C. A., & King, R. M. (1985). The Effect of Timber Harvest on the Fool Creek Watershed, 30 Years Later. *Water Resources Research*, *21*(12), 1915–1922. https://doi.org/10.1029/WR021i012p01915

Troendle, C. A., & King, R. M. (1987). The effect of partial and clearcutting on streamflow at Deadhorse Creek, Colorado. *Journal of Hydrology*, *90*, 145–157.

Troendle, C. A., Wilcox, M. S., Bevenger, G. S., & Porth, L. S. (2001). The Coon Creek Water Yield Augmentation Project: implementation of timber harvesting technology to increase streamflow. *Forest Ecology and Management*, *143*, 179–187.

Ursic, S. J. (1991). Hydrologic effects of two methods of harvesting mature southern pine. *Journal of the American Water Resources Association*, *27*(2), 303–315. https://doi.org/10.1111/j.1752-1688.1991.tb03135.x

Van Haveren, B. P. (1988). A Reevaluation of the Wagon Wheel Gap Forest Watershed Experiment. In *Forest Science* (Vol. 34, Issue 1). https://academic.oup.com/forestscience/article/34/1/208/4641957

Verry, E. S., Lewis, J. R., & Brooks, K. N. (1983). Aspen clearcutting increases snowmelt and storm flow peaks in north central Minnesota. *Journal of the American Water Resources Association*, *19*(1), 59–67. https://doi.org/10.1111/j.1752-1688.1983.tb04557.x

Walsh, P., Jakeman, A., & Thompson, C. (2020). The effects of selective timber harvesting in buffer strips along headwater channels using best management practices on runoff, turbidity and suspended sediment yield in an intensively cut eucalypt forest in southeastern Australia. *Forest Ecology and Management*, *458*. https://doi.org/10.1016/j.foreco.2019.117812

Xiao, L., Robinson, M., & O’Connor, M. (2022). Woodland’s role in natural flood management: Evidence from catchment studies in Britain and Ireland. *Science of the Total Environment*, *813*. https://doi.org/10.1016/j.scitotenv.2021.151877
